# Runx3-mediated Transcriptional Program in Cytotoxic Lymphocytes

**DOI:** 10.1371/journal.pone.0080467

**Published:** 2013-11-13

**Authors:** Joseph Lotem, Ditsa Levanon, Varda Negreanu, Dena Leshkowitz, Gilgi Friedlander, Yoram Groner

**Affiliations:** 1 Department of Molecular Genetics, Weizmann Institute of Science, Rehovot, Israel; 2 Israel National Center for Personalized Medicine Bioinformatics Unit, Weizmann Institute of Science, Rehovot, Israel; Oklahoma Medical Research Foundation, United States of America

## Abstract

The transcription factor Runx3 is highly expressed in CD8^+^ T and NK cytotoxic lymphocytes and is required for their effective activation and proliferation but molecular insights into the transcription program regulated by Runx3 in these cells are still missing. Using Runx3-ChIP-seq and transcriptome analysis of wild type vs. Runx3^-/-^ primary cells we have now identified Runx3-regulated genes in the two cell types at both resting and IL-2-activated states. Runx3-bound genomic regions in both cell types were distantly located relative to gene transcription start sites and were enriched for RUNX and ETS motifs. Bound genomic regions significantly overlapped T-bet and p300-bound enhancer regions in Runx3-expressing Th1 helper cells. Compared to resting cells, IL-2-activated CD8^+^ T and NK cells contain three times more Runx3-regulated genes that are common to both cell types. Functional annotation of shared CD8^+^ T and NK Runx3-regulated genes revealed enrichment for immune-associated terms including lymphocyte activation, proliferation, cytotoxicity, migration and cytokine production, highlighting the role of Runx3 in CD8^+^ T and NK activated cells.

## Introduction

The Runx transcription factor (TF) family consists of 3 highly conserved members (Runx1-3) that function as key regulators of lineage specific gene expression in major developmental pathways [1-3]. Mature CD8^+^ T cells (CD8-TC) and NK cells (NKC) perform similar biological functions. These cytotoxic lymphocytes recognize foreign, infected or tumor cells and following activation undergo interleukin-dependent proliferation. They then kill target cells by releasing perforin and granzyme B-containing granules and cytokines such as tumor necrosis factor (TNF) and interferon gamma (Ifnγ) [4]. 

Runx3 is highly expressed in mature CD8-TC and in NKC and plays an important role in their proliferation and activation [5-7]. Several TFs including Runx family members were shown to participate in development and functions of CD8-TC and NKC but very little is known about the gene targets of these transcription factors [8,9]. The similarity in biological functions of these two cytotoxic cells, their high level of Runx3 and similar defective phenotype upon loss of Runx3 raised the possibility that a common set of Runx3-regulated genes might be involved in their function. Using Runx3 ChIP-seq and transcriptome analysis we identify Runx3-regulated genes in primary CD8-TC and NKC under two different conditions, i.e. at resting and IL-2-activated state. The study established the transcriptional program driven by Runx3 in these cytotoxic lymphocytes, pinpointed many previously unknown Runx3-target genes and singled out a gene subset common to both cell types. This Runx3-regulated cytotoxic cell gene subset is enriched for ontology terms that underscore the importance of Runx3 in regulation of CD8-TC and NKC function. 

## Results

### Resting primary CD8-TC and NKC display similar Runx3 genomic occupancy

**Figure 1:**
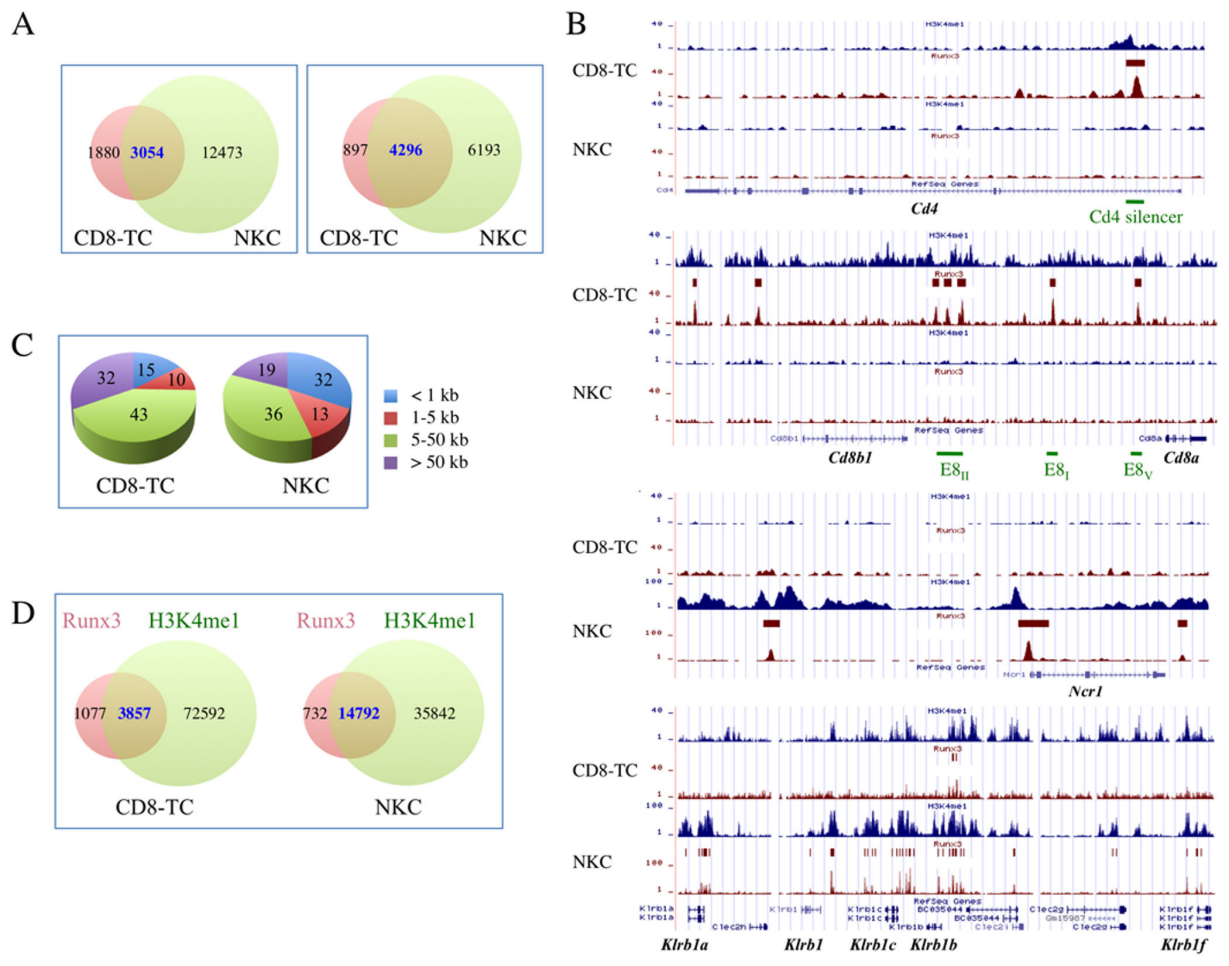
Genome-wide occupancy of Runx3 and H3K4me1 in resting CD8-TC and NKC. (A) Venn diagram summarizing the number of Runx3 ChIP-seq peaks (left panel) and corresponding annotated genes (right panel). (B) Runx3-bound and H3K4me1-marked regions in the Cd4, Cd8, Ncr1 and Klrb1a-Klrb1f loci. Brown and blue tracings reflect the Chip-seq tracing wiggle files uploaded to UCSC Genome Browser mm9 genome assembly of Runx3 and H3K4me1, respectively. Brown rectangles mark Runx3 peaks identified by MACS. Positions of the Cd4 silencer and some of the known Cd8 enhancers are marked in green. (C) Pie chart showing the % of Runx3-bound regions relative to the nearest TSS. (D) Overlap of Runx3-bound and H3K4me1-marked regions.

Runx3 ChIP-seq was conducted using the highly specific in house polyclonal anti-Runx3 antibody Poly-G [10] (Figure S1). Model-based Analysis of ChIP-Seq (MACS) identified 4934 and 15524 Runx3-bound regions in CD8-TC and NKC, respectively, with CD8-TC/NKC occupancy overlap of 62% (Figure 1A left). Association of Runx3-bound regions with annotated genes identified 5193 and 10489 Runx3-bound genes in CD8-TC and NKC, respectively, reflecting an average number of ~1-1.5 Runx3-bound region per gene. The higher number of Runx3-bound genes compared to Runx3-bound regions in resting CD8-TC resulted from shared peaks in the intergenic region separating adjacent genes. Remarkably, ~83% of the Runx3-bound genes in CD8-TC (4296/5193) were bound by Runx3 in NKC (Figure 1A right). This highly similar Runx3 genomic occupancy suggests that although derived from 2 different lineages a common gene subset was Runx3-bound in both cell types. Indeed, using contingency tables we found a significant relationship between Runx3-bound genes in both cell types and their expression above background (p<2.2E-16 in both Pearson Chi-square and Fisher exact tests). 

Despite the similarity in Runx3 occupancy landscape there were bound genes unique to CD8-TC including *Cd4*, silencer in first intron [5] and *Cd8*, enhancers located between *Cd8a* and *Cd8b1* [11] (Figure 1B). Likewise, unique NKC Runx3-bound genes included *Ncr1* encoding the NKC lineage restricted receptor NKp46 [12] (Figure 1B) and many regions in killer-cell lectin-like receptor (Klr) genes (Figure 1B). In contrast, Runx3 occupied only few *Klr* regions in CD8-TC (Figure 1B). These latter findings are consistent with the lower number of killer-like immunoglobulin-like-receptor (KIR) genes expressed in human CD8-TC compared to NKC [13] and underscored the cell-type specific Runx3-binding to lineage-defining genes.

### Runx3-bound regions are remote from transcription start sites and enriched for RUNX and ETS motifs

Analysis of Runx3 occupancy sites relative to transcription start site (TSS) of annotated genes, reveled that 75% and 55% of Runx3 peaks in CD8-TC and NKC, respectively, were located more than 5 kb away from the nearest TSS, and 19%-32%, respectively, were >50 kb away from a TSS (Figure 1C). These results indicated that similar to Runx1 [14,15] and other transcription factors [16], Runx3 regulates a substantial fraction of its gene targets by long-range enhancer-promoter interactions. This notion is supported by the prominent (80-95%) overlap of Runx3 peaks with enhancer-enriched H3K4me1 marked regions in these cells (Figure 1B, D). Nevertheless, ~15% and ~32% of the peaks in CD8-TC and NKC, respectively, were located close to gene TSS (<1 kb upstream or downstream) in regions that could be considered as promoters (Figure 1C). 

**Figure 2:**
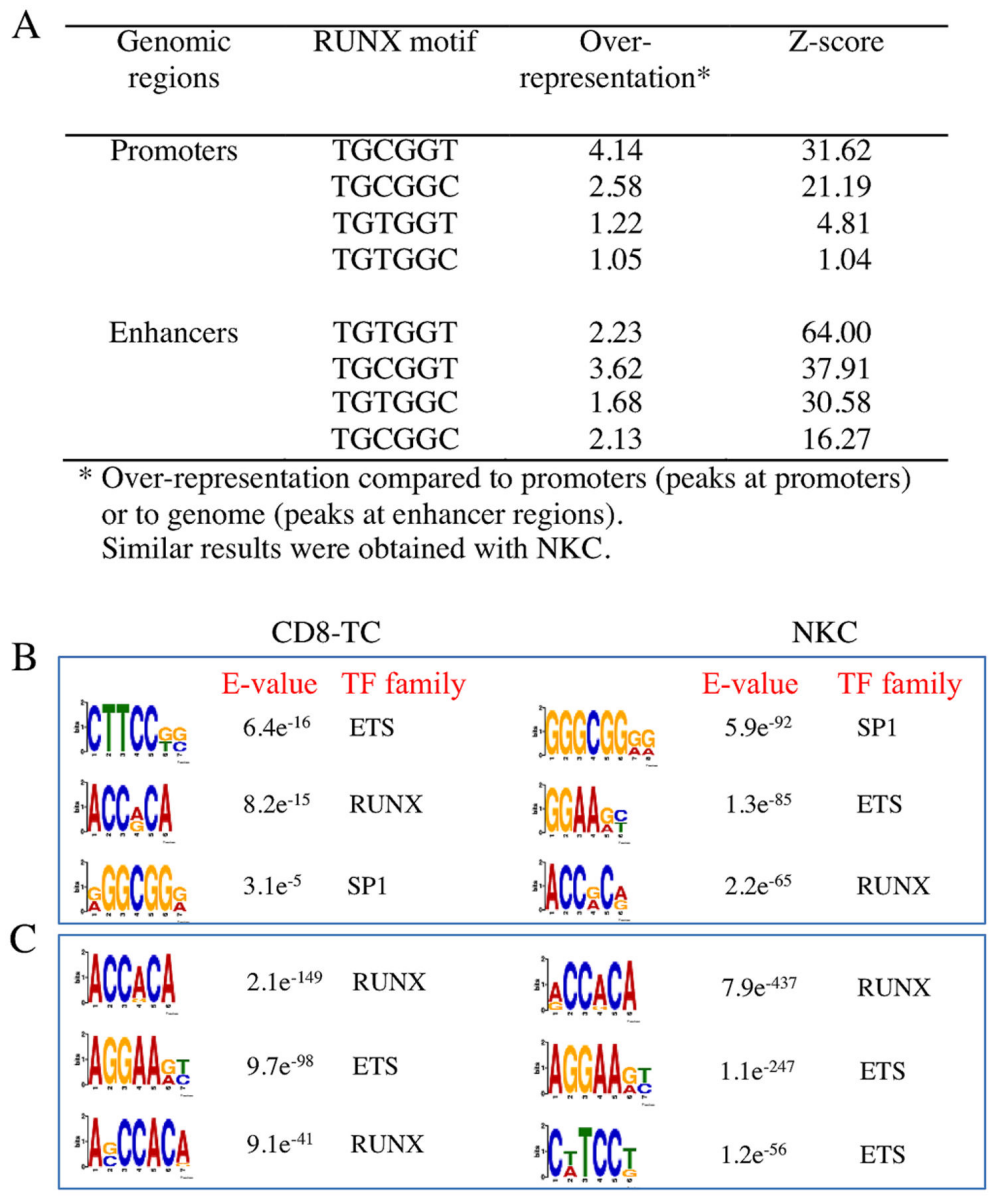
Enriched motifs within Runx3-bound regions in resting cells. (A) Overrepresentation of RUNX motif variants among Runx3-bound regions in CD8-TC. (B and C) Results of de novo motif finding analysis spanning Runx3-bound regions in CD8-TC and NKC. The 3 most enriched motifs in Runx3-bound promoter (B) and enhancer (C) regions are shown.

The DNA sequence TGt/cGGt/c is considered the canonical RUNX motif [17]. We have found that ~90% of Runx3-bound regions in both cell types harbored a RUNX motif. The remote, enhancer regions were enriched for the 4 motif variants compared to genomic background, with the highest Z-score for TGTGGT (Figure 2A), whereas the proximal TSS bound regions were enriched for the TGCGGT and TGCGGC motif variants (Figure 2A). *De novo* motif finding analysis [18] identified enrichment for RUNX and ETS TF family motifs among Runx3-bound regions, as well as enrichment at promoter regions for the common SP1 motif (Figure 2B, C). These data indicate that Runx3 binding to its genomic motif in collaboration with Ets TF is the major event driving Runx3 transcriptional program in CD8-TC and NKC at the resting state. Indeed ChIP-seq-based prediction analysis of biological functions [19] revealed enrichment for ontology terms of immunity-associated functions, including T cell activation, cytokine production, decreased lymphocyte numbers, IL-2-mediated signaling, chemokine signaling and NKC mediated cytotoxicity (Table S1).

### Runx3-regulated genes in resting CD8-TC and NKC

Transcriptome analysis of Runx3^-/-^ vs. WT cells detected 609 and 1243 differentially expressed (i.e. Runx3-responsive) genes in resting CD8-TC and NKC, respectively, with ~60% and ~40% of these genes being down-regulated and up-regulated, respectively, in both cell types (Table S2). Cross analysis of Runx3-responsive with RUNX-motif-bearing Runx3-bound genes (thereafter defined as Runx-regulated) revealed a significant overlap (p<2.2E-16 in both Pearson Chi-square and Fisher exact tests). Specifically, the cross analysis identified 231 and 818 Runx3-regulated genes in CD8-TC and NKC, respectively (Table S3), comprising 38% and 68% of the Runx3-responsive genes in CD8-TC and NKC, respectively. This result suggests that at resting state a larger fraction of Runx3-responsive genes in NKC is directly regulated by Runx3 compared to CD8-TC. 

Only a handful of genes considered as Runx3-targets were previously reported to be affected by loss of Runx3 during CD8-TC development, including *Cd4* [5,6], *Zbtb7b*/*ThPOK* [20] and *Itgae/*CD103 [21,22]. *Cd4* and *Itgae* showed increased and decreased expression, respectively, upon loss of Runx3 (Tables S2, S3). *Itgae* was also Runx3-regulated in resting NKC (Tables S2, S3). There were 38 Runx3-regulated genes common to resting CD8-TC and NKC (Table S4), significantly higher (p=2.8E-16) than 11 common genes expected by chance, using the method described by Smid et al, 2003 [23].

### Activation of CD8-TC and NKC by IL-2 alters Runx3 genomic occupancy

**Figure 3:**
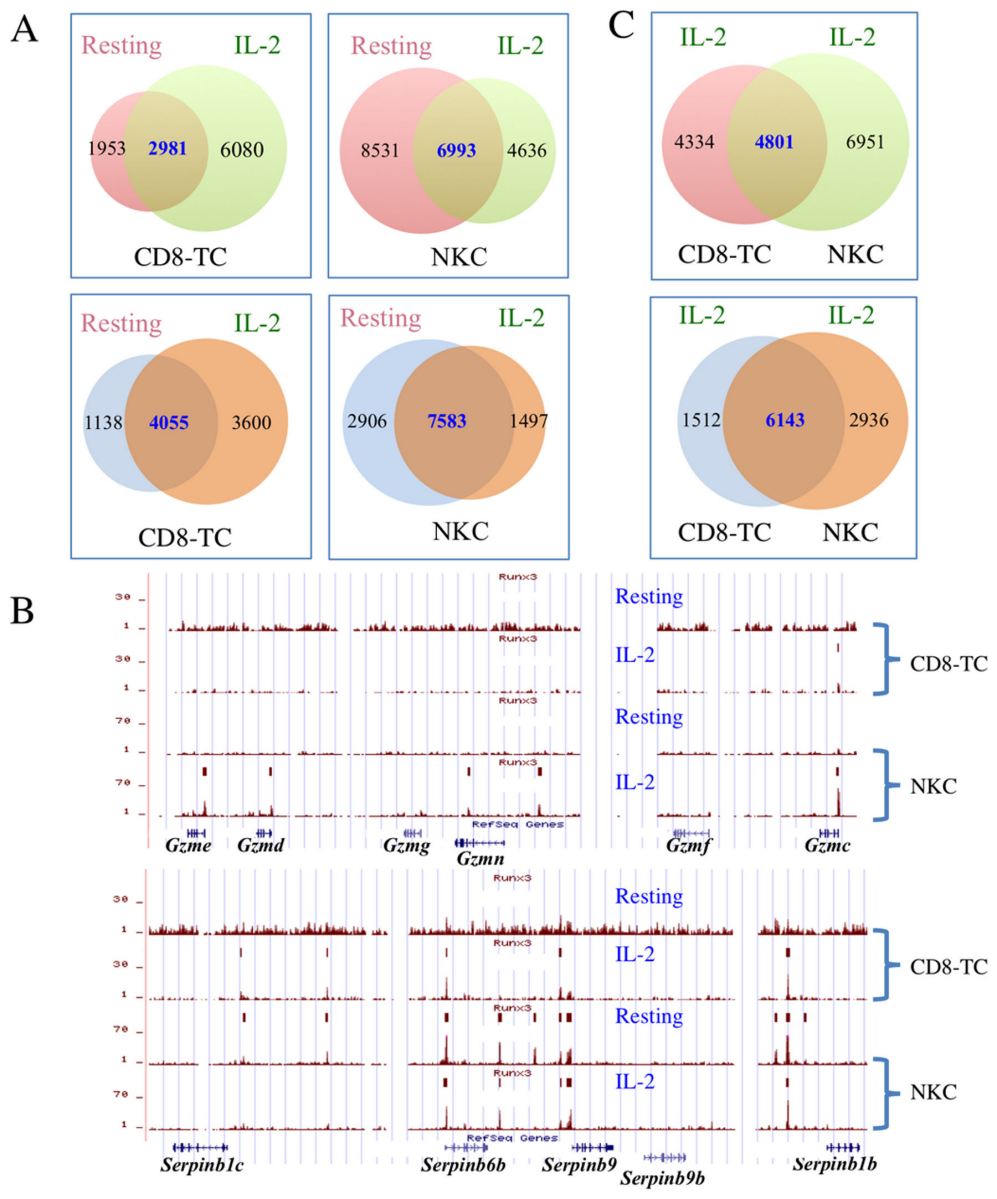
Runx3-bound regions and their corresponding annotated genes in IL-2-activated compared to resting CD8-TC and NKC. (A) Overlap of Runx3-bound (upper panels) regions or their corresponding genes (lower panels) in resting and IL-2-activated CD8-TC (left) or NKC (right). The genes corresponding to Runx3-bound regions were derived using GREAT [19]. (B) Recruitment of Runx3 to de novo IL-2-activated regions in Gzme-Gzmc (top) and Serpinb1c-Serpinb1b (bottom) loci, in NKC and CD8-TC, respectively. Brown tracing and rectangles represent Runx3 ChIP-seq wiggle files and the positions of Runx3 peaks, respectively, as in Figure 1. Note that some de novo Runx3-bound regions in Serpin genes in IL-2-activated CD8-TC appear to bind Runx3 in resting CD8-TC, but these regions are not scored by MACS as peaks in resting CD8-TC due to the higher background. (C) Overlap of Runx3-bound regions (upper panel) and their corresponding genes (lower panel) in IL-2-activated CD8-TC and NKC.

Following encounter with foreign or infected cells, both cell types become activated and proliferate extensively in a cytokine-dependent manner. In view of their defective proliferation and maturation/activation phenotypes caused by Runx3 loss, we evaluated the impact of IL-2-induced activation on Runx3 binding as compared to the resting cells pattern. ChIP-seq analysis of IL-2-activated cells revealed changes in the number of Runx3-boud regions compared to their resting state (Figure 3A upper panels). IL-2-induction was associated with recruitment of Runx3 to 3600 and 1497 *de novo* genes in CD8-TC and NKC, respectively, and with loss of Runx3 binding to 1138 and 2906 genes, respectively (Figure 3A lower panels). For example, Runx3 was recruited to several *de novo* sites in regions spanning the *Gzme-Gzmc* and *Serpinb1c*-*Serpinb1b* loci in NKC and CD8-TC, respectively (Figure 3B), demonstrating the dynamic nature of Runx3 binding during IL-2-induced activation. The high overlap in Runx3 occupancy observed in resting NKC and CD8-TC was maintained in their activated state. In IL-2-activated CD8-TC, more than 50% of Runx3-bound regions (4801 out of 9135) overlapped with those of IL-2-activated NKC (Figure 3C upper panel) and significantly higher overlap (~80%; 6143 out of 7655) was noted in Runx3-binding to annotated genes (Figure 3C lower panel). Thus, despite the activation-induced changes in number and characteristics of the Runx3-bound genes the degree of overlap between CD8-TC and NKC was largely maintained at activated state. 

**Figure 4:**
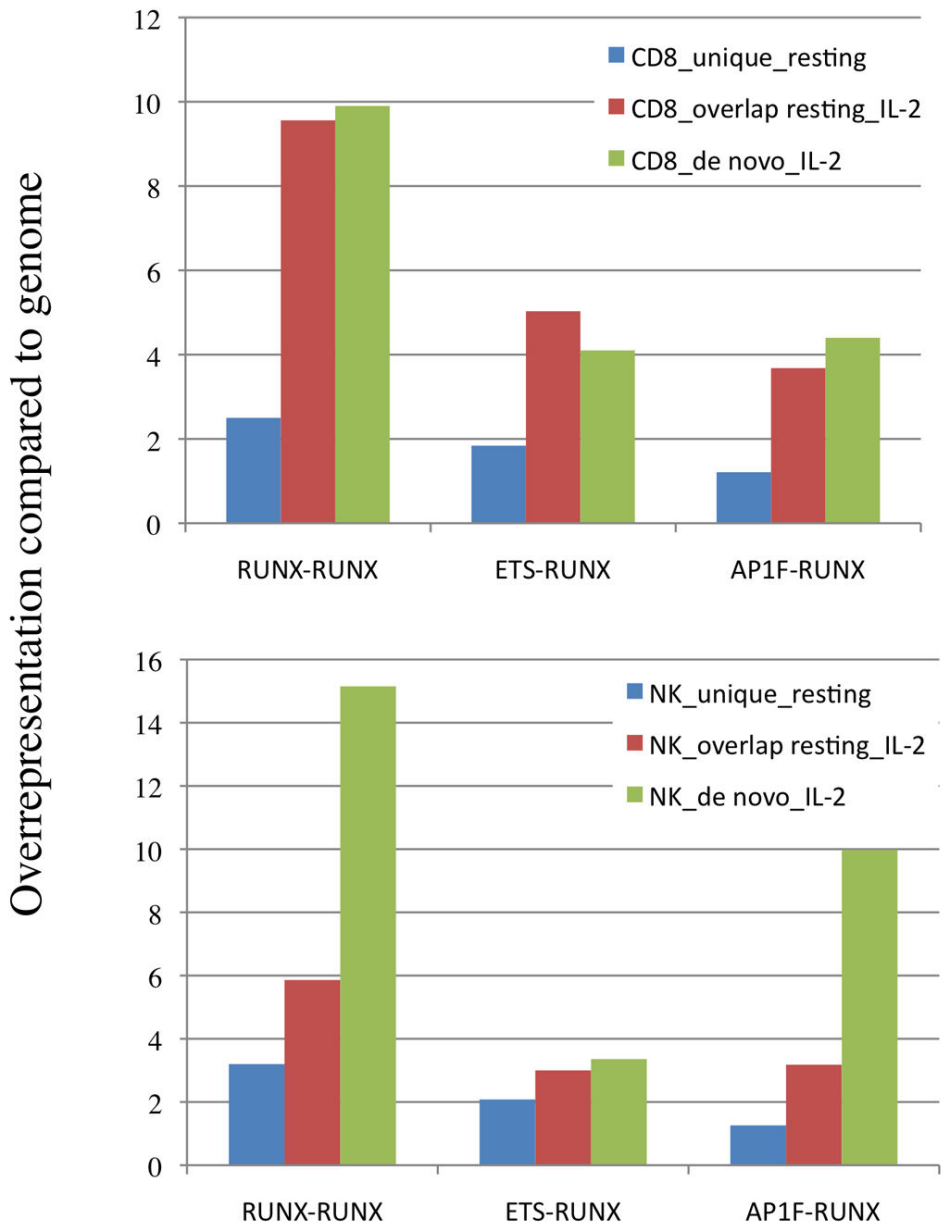
Increased enrichment of RUNX-RUNX, ETS-RUNX and AP1-RUNX modules in uniquely Runx3-bound regions of IL-2-activated compared to resting cells. Histograms show the degree of enrichment compared to genome of the above modules in Runx3 peaks unique to resting cells, overlapping peaks in resting and IL-2-activated cells and de novo peaks unique to IL-2-activated cells.

As in resting state, more than 90% of Runx3 peaks in IL-2 activated cells contained a canonical RUNX motif and motif finding analysis [18] revealed RUNX and ETS as the most enriched motifs in Runx3-bound regions (Figure S2A, B). Interestingly, an AP-1 TF family motif was enriched in Runx3-bound enhancer regions in IL-2-activated NKC (Figure S2B) and RUNX-RUNX, ETS-RUNX and AP-1-RUNX modules were enriched in bound enhancer regions of both cell types (Figure S2C). These modules were significantly more enriched in the *de-novo-*IL-2 Runx3-bound regions as compared to resting cells (Figure 4). Together, these results suggest that Ets and AP-1 collaborate with Runx3 in transcription regulation at CD8-TC and NKC activated state. 

### IL-2 activated CD8-TC and NKC Runx3-occupied regions overlap with T-helper-specific enhancers

**Figure 5:**
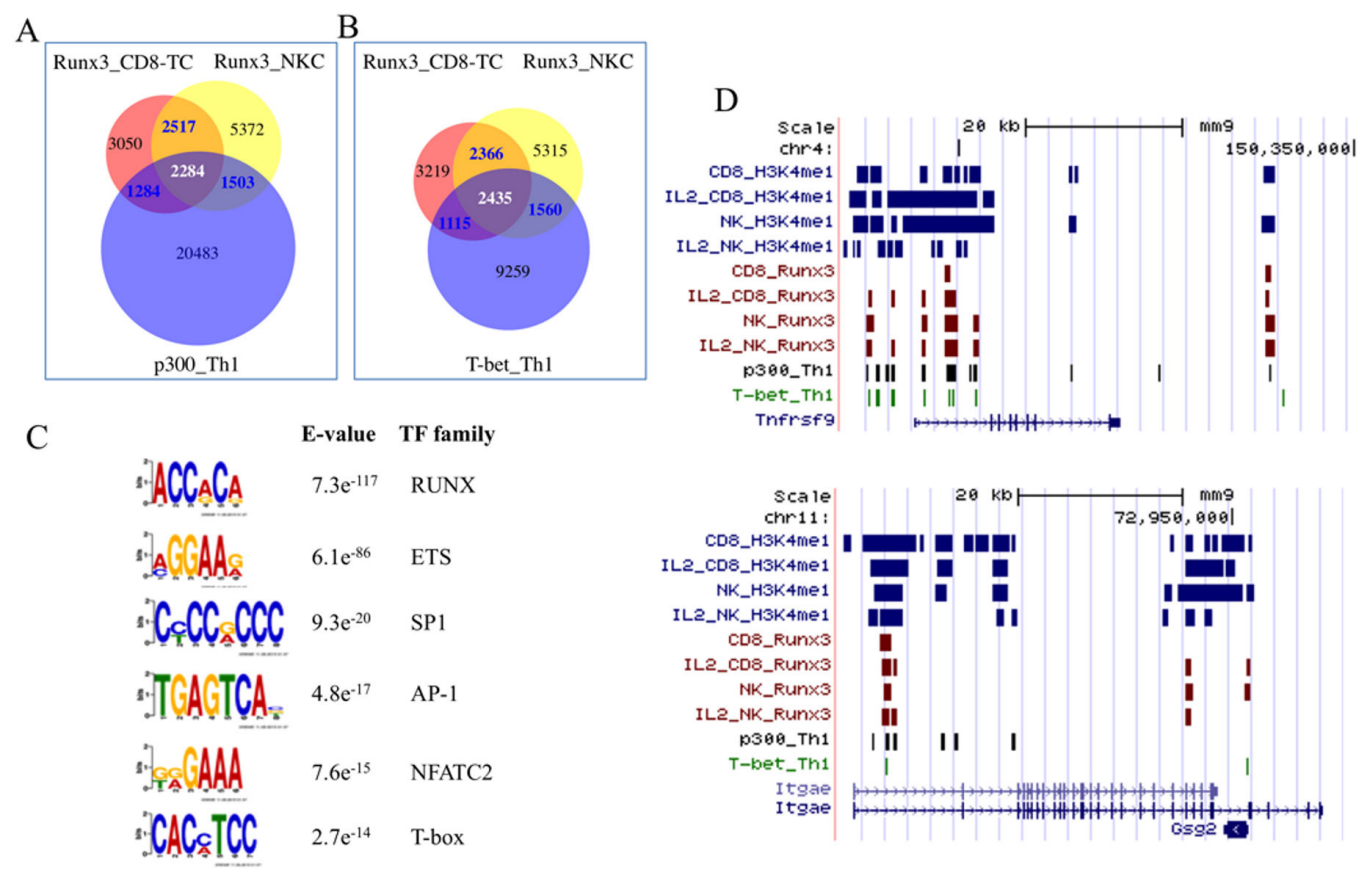
Overlap of Runx3-bound regions in IL-2-activated CD8-TC and NKC with p300 and T-bet-bound regions in Th1 cells. (A and B) Overlap of Runx3-bound regions in CD8-TC/NKC with p300 and T-bet peaks, respectively, in Th1 cells. The locations of Th1 p300 [24] and T-bet [25] peaks were obtained from processed data in public repository and these papers. (C) De novo found TF motif enrichment of overlapping CD8-TC/NKC Runx3-bound and Th1 T-bet-bound regions. (D) Examples of gene loci with overlap Runx3-bound and H3K4me1-marked regions in resting or IL-2-activated CD8-TC and NKC with p300 and T-bet-bound regions in Th1 cells.

The observation that Runx3 occupies a large number of common genomic regions remote from gene TSS, suggested that these regions might function as CD8-TC/NKC enhancers. This possibility was supported by the finding of marked overlap (~50%) of IL-2-activated CD8-TC/NKC Runx3-bound regions with previously reported p300-bound [24] and T-bet TF-bound [25] regions in Th1 activated CD4^+^ T cells (Figure 5 A, B), manifested in a 65-75% overlap of the corresponding annotated genes. 

*De novo* motif finding analysis [18] revealed that these Runx3/T-bet overlapping regions were enriched for RUNX, ETS, AP-1, NFATC and T-box/ Eomes motifs (Figure 5C), suggesting cooperation of Runx3 and these other TFs in gene expression regulation of IL-2-activated CD8-TC, NKC and Th1. Runx3 expression in resting CD4^+^ T cells is quite low but induced in a T-bet-dependent manner upon activation and differentiation along the Th1 lineage [26,27]. Runx3 and T-bet then collaborate in regulating expression of *Ifnγ* and *Il4* in Th1 cells [26]. Runx3 also cooperates with T-bet and Eomes TFs in regulating CD8-TC maturation and function [7] and these TFs play role in maturation and function of NKC [9,28] and Th1 cells. It thus appears that Runx3 occupies a large number of enhancer regions to regulate CD8-TC/NKC gene expression in cooperation with additional lineage specific TF that also function in Th1 cells (Figure 5D). 

### Runx3-regulated genes in IL-2-activated CD8-TC and NKC

**Figure 6:**
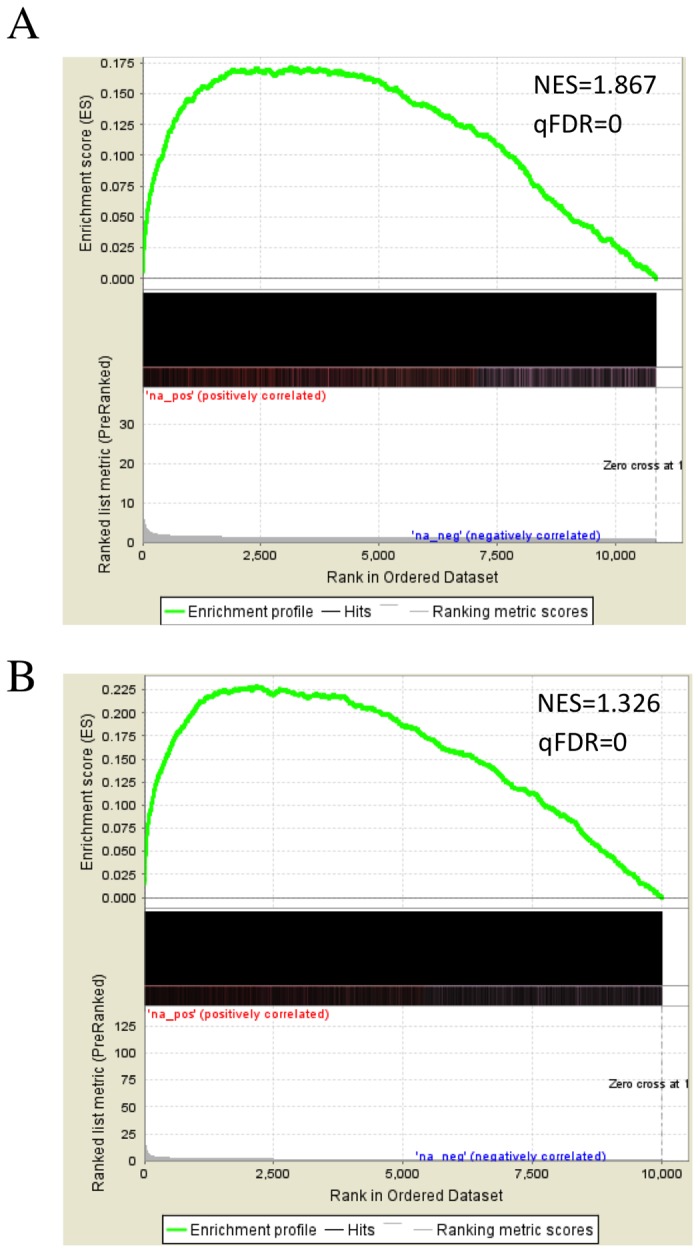
GSEA analyzed relationship of differential gene expression in WT/Runx3^-/-^ cells and Runx3-bound genes in IL-2-activated CD8-TC and NKC. (A and B) All microarray genes were pre-ranked according to absolute linear fold changes of WT vs. Runx3^-/-^ and statistical enrichment of Runx3-bound genes within the ranked list was evaluated in CD8-TC (upper panel) and NKC (lower panel). NES, normalized enrichment score.

Transcriptome analysis revealed 501 and 3060 Runx3-responsive genes (Runx3^-/-^ vs. WT) in IL-2 activated CD8-TC and NKC, respectively (table S2), with a similar frequency of up- or down-regulated genes in both cell types (Table S2, Figure S3). Gene Set Enrichment Analysis (GSEA) [29] revealed a strong relationship between differentially expressed genes and Runx3 binding in both types of IL-2-activated cells (Figure 6). Cross analysis of Runx3-responsive with motif-bearing Runx3-bound genes revealed 341 and 1848 Runx3-regulated genes in IL-2-activated CD8-TC and NKC, respectively (table S3). Using quantitative PCR (qPCR) analysis we further validated Runx3 responsiveness of 7 genes in IL-2-activated CD8-TC (Figure S4). 

**Figure 7:**
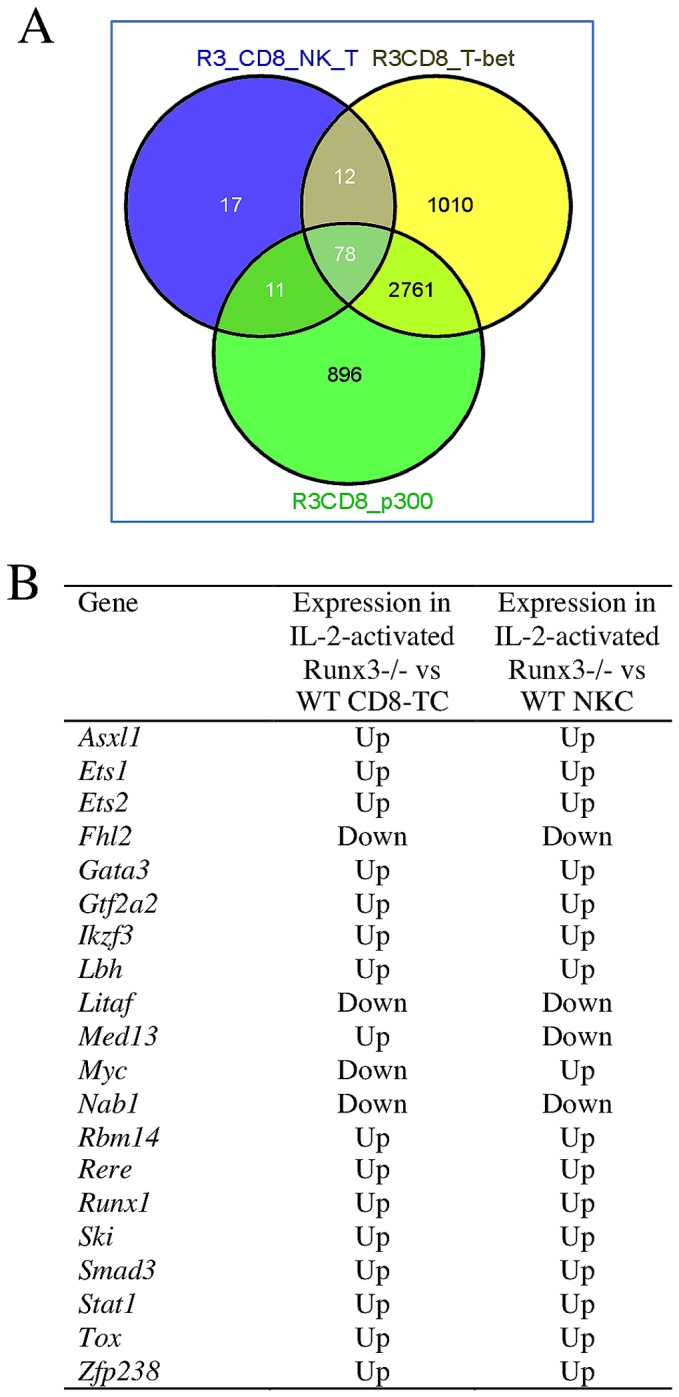
Common Runx3-regulated genes in IL-2-activated CD8-TC/NKC and T-bet/p300 bound genes in Th1. (A) The majority of the 118 common Runx3-regulated genes in IL-2-activated CD8-TC and NKC (R3_CD8_NK_T) harbor overlapping T-bet (R3CD8_Tbet) and p300 (R3_CD8_p300) bound regions in Th1 cells. (B) Transcription factors/regulators that are common Runx3-regulated genes in IL-2-activated CD8-TC and NKC.

Of the Runx3-regulated genes identified in IL-2 activated CD8-TC and NKC, a significant number (118) were common to both cell types (p=2.7E-41), compared to 37 common genes expected by chance (Figure 7A, table S4). This observation is consistent with the possibility that these genes were involved in producing the CD8-TC/NKC common phenotypic features in Runx3^-/-^ cells. The finding that loss of Runx3 affected 78% (92 of 118) of these genes in the same direction (up- or down-regulated) in both cell types (table S4) supports this possibility. Interestingly, 78 of the common Runx3-regulated genes (~66%) harbored Runx3-bound regions that overlapped with both T-bet and p300-bound regions in Runx3-expressing Th1 cells (Figure 7A) and 20 of them are TFs/regulators known to be involved in various aspects of hematopoiesis and immunity (Figure 7B). This frequency of TFs among the common Runx3-regulated genes in IL-2-activated cells is significantly higher (p=0.004126 and 0.007799 in Pearson Chi-square and Fisher exact tests, respectively) than TF frequency in mouse genome (~6.8%). Functional annotation of the 118 common Runx3-regulated genes in activated CD8-TC and NKC using Ingenuity Systems Pathway Analysis software (http://www.ingenuity.com), revealed enrichment for ontology terms including cellular growth and proliferation, cellular development, cell death and inflammatory response (Figure S5). Taken together, the results suggest that Runx3 plays an important regulatory role in IL-2-induced CD8-TC/NKC activation in inflammatory processes. 

### Runx3-regulated genes and IL-2-activated CD8-TC and NKC proliferation

**Figure 8:**
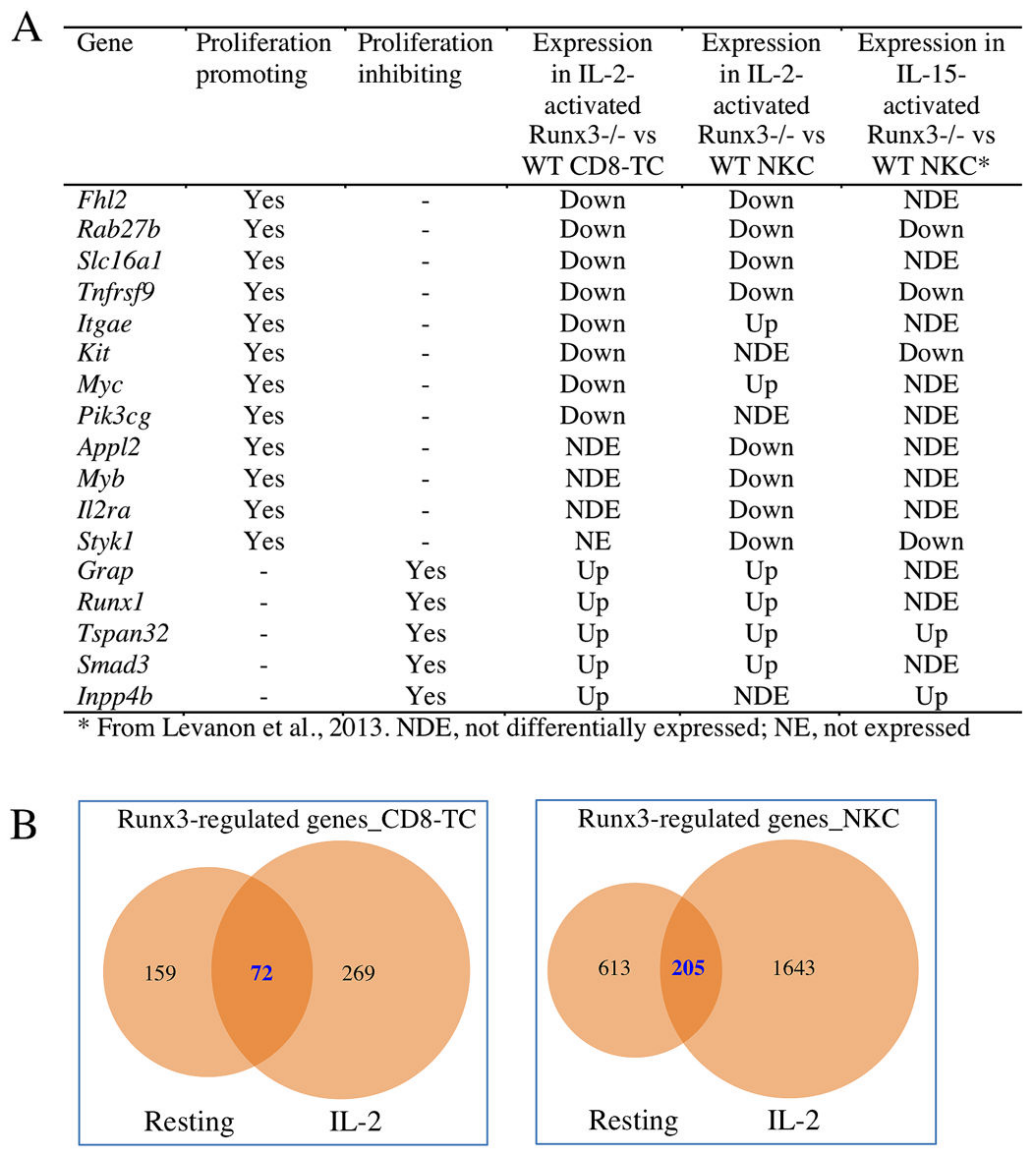
Proliferation-control Runx3-target genes in activated CD8-TC/NKC and common Runx3-regulated genes in resting and IL-2 activated cells. (A) Runx3-regulated genes that may be involved in the defective proliferation of IL-2-activated Runx3^-/-^ CD8-TC and NKC. (B) Comparisons of common Runx3-regulated genes in resting and IL-2-activated CD8-TC (left panel) or in resting and IL-2-activated NKC (right panel) reveal 72 and 205 common targets, respectively.

A number of Runx3-regulated genes that could contribute to the impaired proliferation phenotype in IL-2-activated Runx3^-/-^ CD8-TC and/or NKC are described in Figure 8A. Upon loss of Runx3 a number of key proliferation-promoting genes including *Fhl2* [30], *Rab27b* [31], *Slc16a1* [32], *Tnfrsf9* [33], *Itgae* [34], *Kit* [35], *Myc* [36], *Pik3cg* [37], *Appl2* [38], *Myb* [39], *Il2ra* and *Styk1* [40] were down-regulated along with up-regulation of the several proliferation inhibitors such as *Grap* [41], *Runx1* [42], *Smad3* [43], *Tspan32* [44] and *Inpp4b* [45]. Several of these genes encode cell surface receptors and/or molecules that transmit either proliferation or anti-proliferation signals (Il2ra, Tnfrsf9, Kit, Styk1, Itgae, Slc16a1, Tspan32), others encode signaling/adaptor molecules (Pik3cg, Appl2, Rab27b, Grap, Inpp4b, Smad3) and the rest are TFs involved in proliferation control (Myc, Myb, Fhl2, Runx1). It thus appears that Runx3 regulates expression of multiple proliferation-regulating genes whose activity is exerted at major cellular compartments that transmit signals from the cell surface membrane to the nucleus. 

### Runx3-regulated genes in resting compared to IL-2-activated CD8-TC and NKC

Combined ChIP-seq and gene expression analyses revealed that the number of Runx3-regulated genes in IL-2 activated CD8-TC and NKC was 1.5- and 2.2-fold, respectively, higher than at the cell resting state (table S3). Of the Runx3-regulated genes in resting CD8-TC and NKC, 72 and 205 genes, respectively, were also found in IL2-activated cells (Figure 8B). These numbers (72 and 205) are significantly higher (p=2.3E-291 and p=4.3E-61, in CD8-TC and NKC, respectively) than 4 (CD8-TC) and 70 (NKC) common genes expected by chance. Overall the Runx3-mediated CD8-TC/NKC transcriptional program had higher overlap between IL2-activated cells compared to their resting state, reflected in number of shared Runx3-regulated genes and the higher proportion of similarly affected genes upon loss of Runx3 (table S4). 

## Discussion

T cells develop from CLPs via Notch1-Bcl11b-dependent pathway [46] and lack of Notch1 signaling, even after acquisition of a T cell fate, results in a change to a NKC fate [47]. Runx3 expression is induced during development of both CD8-TC [5,6,48] and NKC (unpublished data) and plays a role in regulating certain immune-associated functions in these cytotoxic cells including proliferation and expression of various maturation and activation-associated markers [6,7,21,22,49,50]. Because NK and T cells also show close similarity at the transcriptome level [51], we hypothesized that Runx3-dependent transcriptional programs in these two different cell types will have significant degree of common characteristics. 

### Similarity in CD8-TC and NKC genomic occupancy by Runx3

Runx3 occupies thousands of genomic loci in resting and IL-2-activated CD8-TC and NKC, reflecting a common property of many TFs, including the other two RUNX family members Runx1 [15,52] and Runx2 [53]. About 80% of Runx3-bound genes in CD8-TC overlapped those in NKC. The majority of Runx3-bound regions were distant from TSS and significantly overlapped with p300, and T-bet-bound enhancer regions in Runx3-expressing Th1 cells. These findings are compatible with the possibility that Runx3 plays an important role in regulating expression of the CD8-TC/NKC common genes as well as genes in Th1 cells via the shared enhancers.

### Ets-TFs emerged as a key Runx3 cooperator in driving the CD8-TC/NKC transcriptional program

About 90% of Runx3 peaks in resting and IL-2-activated CD8-TC and NKC harbor at least one RUNX motif that conforms to the canonical DNA sequence TGt/cGGt/c. Interestingly, the variant RUNX motifs were not distributed evenly in Runx3 peaks located at different genomic regions, rather the TGCGGt/c and TGTGGt/c variants were more prevalent at promoter and enhancer regions, respectively. These results indicated that Runx3 binds to DNA directly to canonical RUNX motifs. Indeed, *de novo* motif finding revealed that the most enriched motifs corresponded to these variant canonical RUNX motifs, as was also found for Runx1-bound regions in megakaryocytes [54] and for Runx3 peaks of *in vivo* IL-15-activated NK cells.

In addition to RUNX, Runx3-bound promoter and enhancer regions are enriched for ETS family motifs and *de novo* occupied regions that are unique to IL-2-activated cells are enriched for RUNX-RUNX and ETS-RUNX modules. These findings suggested that Runx3 and Ets family TFs cooperate in regulating common target genes in CD8-TC and NKC. This possibility is supported by observations that Runx family TFs [55] including Runx1 [15] and Runx2 [56,57] collaborate with ETS family members in other cell types. Of particular relevance to the RUNX/ETS cooperation notion is the mouse ENCODE project finding [58] that RUNX and ETS were among the most enriched motifs specifically in enhancer regions of hematopoietic organs including spleen, bone marrow and fetal liver. 

Various Ets family TFs were reported to participate in development and function of both CD8-TC and NKC [59-61]. We have now found that Runx3 either negatively or positively regulates several *Ets* family genes. For example, Runx3 repressed *Ets1* and *Ets2* in IL-2-activated CD8-TC and NKC (Figure 7B, tables S2, S3) as well as *Etv3* and *Etv6* in IL-2-activated NKC (tables S2, S3). Runx3 also repressed *Ets2* and *Elf1* in resting CD8-TC and NKC, respectively (tables S2, S3) and *Ets2* in IL-15-activated NKC (GEO (http://www.ncbi.nlm.nih.gov/geo/) GSE50131). On the other hand, Runx3 enhanced expression of the *Ets* family genes *Fli1, Gabpa* and *Gabpb1* in IL-2-activated NKC (tables S2, S3). Furthermore, in CD8-TC Ets1 transcriptionally activated *Runx3* expression [60]. Collectively, these findings suggest that Runx3 and Ets TFs not only cooperate in gene expression regulation in these cell lineages, but may also cross-regulate each other. This latter occurrence might have far reaching effects on cell biological characteristics upon loss of either of these TFs. We also noted that *Gata3* and *Runx1* are among transcriptionally repressed Runx3-regulated genes in IL-2-activated CD8-TC and NKC (Figure 7B, tables S3, S4), in line with the ability of Gata3 and Runx3 to negatively regulate each other during T cell development [62,63] and the ability of Runx members to cross-regulate each other and their mutually exclusive expression in various cell systems [1,64].

### AP-1 motif enrichment and AP-1/Runx3 co-occupancy in IL-2-activated cells

Runx3-bound enhancer regions in IL-2-activated NKC were enriched for the AP-1 TF family motif and the AP-1-RUNX module was highly enriched in the *de novo* Runx3-occupied regions unique to IL-2-activated CD8-TC and NKC compared to resting cells. Interestingly, p300-bound enhancer regions in T-helper cells are also enriched for both RUNX and AP-1 motifs [24] and so are Runx1-bound regions in differentiating megakaryocytic cells [15] and hemogenic endothelium cells [65]. These results suggest that Runx3 and AP-1 might collaborate and play a significant role in transcription regulation during IL-2-induced activation of CD8-TC and NKC. Such collaboration occurs between RUNX1 and AP-1 during differentiation of megakaryocytic cells [15] and between Runx2 and AP-1 in regulating expression of *Mmp13* in osteoblasts [66]. This scenario is supported by the induction and requirement of AP-1 transcriptional activity for TCR/CD28-induced activation and IL-2 expression [67]. 

AP-1 regulates expression of human *NKG2D* (mouse *Klrk1*) in both CD8-TC and NKC [68]. In both cell types Runx3 occupies *Klrk1*, which encodes a co-stimulatory receptor for CD8-TC [69], and *Klrk1* is among Runx3-regulated genes in IL-2-activated CD8-TC that are down regulated in Runx3^-/-^ cells (table S3). *Fhl2*, which is Runx3-bound and down regulated in Runx3^-/-^ IL2-activated CD8-TC and NKC (tables S3, S4), is a known transcriptional co-activator of AP-1 [70]. Fhl2 interacts with Runx2 and increases its transcriptional activity [71], while Runx1 and Traf6 transcriptionally activate Fhl2 via the RUNX response element in its promoter [72]. Together, these results suggest that Runx3-dependent transcriptional activation of *Fhl2* during in IL-2-activated CD8-TC and NKC might facilitate regulation of other genes by the combined activity of Runx3 and AP-1.

### Runx3 regulates common genes in CD8-TC/NKC resting and IL-2-activated states

The landscape of Runx3 genomic binding in CD8-TC/NKC suggested its involvement in regulating activation, proliferation, migration and cytotoxicity. We have identified a Runx3-regulated gene subset common to CD8-TC and NKC and found a 3-fold higher number of common genes at IL-2-activated compared to resting state. This finding corresponds with the more prominent Runx3^-/-^ phenotype of IL-2-activated cells and *in vivo* IL-15-activated NKC compared to resting state. 

A number of Runx3-regulated genes identified in IL-2-activated CD8-TC and/or NKC could be involved in the attenuated proliferation of IL-2-activated Runx3^-/-^ versus WT cells. It is interesting to note that six of these genes were also identified as Runx3-regulated in *in vivo* IL-15-activated NKC (Figure 8A) and their expression in Runx3^-/-^ cells was affected in the same direction as in IL-2-activated CD8-TC and/or NKC (Figure 8A). Thus, while IL-2 and IL-15 transcriptional programs in NKC may be somewhat different (unpublished data), it appears that a subset of Runx3-regulated genes contributes to the attenuated proliferation of Runx3^-/-^ NKC in response to both cytokines. Of particular interest are 3 Runx3-regulated genes, *Rab27b*, *Tnfrsf9* and *Tspan32*, of which the former two proliferation-promoting genes are down regulated and the latter proliferation-inhibitor is up regulated upon loss of Runx3 in activated CD8-TC and NKC. *Rab27b* encodes a small GTPase that regulates vesicle trafficking in the exocytic secretory pathway in various cell types and when overexpressed it promotes G1/S transition and cell proliferation [31]. Although no information is available about *Rab27b* role in CD8-TC or NKC, its reduced expression in Runx3-deficient cells might have an impact on their proliferation. *Tnfrsf9* encodes a TNF family receptor whose ligation in T cells activates the PI3K/Akt pathway, which subsequently results in highly concentrated depots of PI3K and Akt in close proximity to TCR signaling units [73] and stimulates their proliferation [33]. In NKC, Tnfrsf9 promotes IL-2/15-induced proliferation [74], which involves MAPK signaling [75]. Tspan32 is a member of the tetraspanin family of cell membrane proteins that interact with key leukocyte receptors and other surface proteins and organize them into functional microdomains that activate downstream signaling pathways [76]. Although the nature of the Tspan32-induced signaling pathway(s) is not known, the enhanced lymphocyte-proliferation phenotype in Tspan32-deficient cells [44] suggests that it transmits negative signals for proliferation. 

Finally, it should be noted that gene expression during activation, proliferation and differentiation is a highly dynamic temporal process and so is the pattern of Runx3 genomic binding. Therefore, it is quite possible that a different set of Runx3-regulated genes might be identified at other time windows after induction of cell activation than those we have analyzed in the present experiments. 

## Conclusions

The cytotoxic lymphocytes CD8-TC and NKC constitute an important immune defense system against infected and tumor cells. Runx3 TF is highly expressed in these cells and its loss affects their development and functions. Like other TFs that participate in their development, the transcriptional program of Runx3 in CD8-TC and NKC was largely unknown and here we provide a whole-genome insight into its role in the physiology of these cells. We have identified hundreds of previously unknown Runx3 target-genes; singled out a set of Runx3 targets common to both cell types and highlighted its potential collaborating TFs and the special importance of Runx3-regulated genes in cytokine-induced proliferation of activated cells. The data constitute a comprehensive resource for further studies with other TFs that collaborate with Runx3 in regulation of CD8-TC and NKC development and activation.

## Materials and Methods

### Cells and cell culture

CD8-TC were isolated from spleen of WT ICR mice using anti-CD8 magnetic beads according to manufacturer instructions (BD Biosciences) yielding a highly enriched population (~95%) of CD3^+^CD8-TC. In Runx3-/- mice, the anti-CD8 separated spleen cells contained ~20% of CD8^+^CD11c^+^ dendritic cells, which were removed by FACS sorting. NKC from WT and Runx3-/- spleens were enriched by negative selection using NK isolation kit (R&D systems Inc., USA) and FACS sorted to obtain DX5^+^ cells. Freshly isolated cells were defined as resting CD8-TC and NKC. CD8-TC (8x10^5^ cells/ml) were activated for 2 days using anti-CD3e (eBioscience, Inc., USA, 1 mg/ml) coated plates plus soluble anti-CD28 (eBioscience, Inc., USA, 1 mg/ml) as described [7]. Cells were then diluted to 5x10^3^ cells/ml and cultured for 4 days in the presence of 100u/ml recombinant human recombinant interleukin 2 (IL-2) (Biological Industries, Beit Haemek, Israel). NKC were activated by culture for 7 days in the presence of 1000 units/ml IL-2 or mouse IL-15 (Peprotec, Israel). At the end of culture >98% of cells were CD3^+^CD8-TC and NKp46^+^ NKC. These experiments were carried out in strict accordance with the recommendations in the Guide for the Care and Use of Laboratory Animals of the National Institutes of Health. The protocol was approved by the Committee on the Ethics of Animal Experiments of the Weizmann Institute of Science (Permit Number: 01190113-2). All surgery was performed under sodium pentobarbital anesthesia, and all efforts were made to minimize suffering.

### ChIP-seq processing and data analysis

ChIP was performed essentially as described [15]. Briefly, cross-linked chromatin from ~3-5x10^7^ resting or IL-2-activated CD8-TC or NKC, was fragmented to an average size of ~200-400 bp by 22-40 cycles of sonication (30 sec each) in 15 ml tubes using the Bioraprtor UCD-200 sonicator (Diagenod, US). For ChIP, 40 ul of anti-RUNX3 [10] or anti-monomethyl-Histone H3 (Lys4) (Millipore, US) antibodies were added to diluted and fragmented chromatin. Rabbit non-immune serum (NIS) was used as a control. DNA was purified using QIAquick spin columns (QIAGEN, US) and sequenced with Illumina genome analyzer IIx, according to manufacturer instructions. Two biological repeats were conducted and separately sequenced. For ChIP-seq analysis, Illumina sequencing short reads (40-42 bp) were aligned to the mouse genome (mm9) using the ELAND program (Illumina) and only uniquely aligned reads were further analyzed. Peaks were determined with MACS [77] version 1.4 on joined reads from both biological repeats, using NIS as control and scaling the large dataset to the small dataset. Peak calling by MACS is done using a dynamic Poisson distribution to effectively capture local genome sequence biases, which in the shown analysis were computed using the control (NIS) background. The numbers of uniquely aligned reads after MACS filtration for NIS, Runx3 and H3K4me1 were: 30.9, 32.8 and 32.5 million, respectively, in resting CD8-TC; 26.3, 25.5 and 29.8 million, respectively, in resting NKC; 6.2, 7.6 and 12.5 million, respectively, in IL-2-activated CD8-TC; and 11.2, 18.8 and 11.5 million, respectively, in IL-2-activated NKC. Genomic Regions Enrichment of Annotations Tool (GREAT) algorithm [19] with default parameters was used to determine the genes corresponding to Runx3-bound peaks and the distance of these peaks from TSS of RefSeq genes. The coordinates of the p300 and T-bet bound regions were obtained from processed data in public repository and the relevant references 24] and [25, respectively. Overlapping peaks between different treatments and/or cell types were determined with Genomatix Genome Analyzer (http://www.genomatix.de/solutions/genomatix-genome-analyzer.html) using the Bed-file tool at default parameters. 

### Enriched motifs in Runx3 peaks

To find peaks with the consensus RUNX motif TGt/cGGt/c we used CisGenome (http://www.biostat.jhsph.edu/~hji/cisgenome/). Overrepresentation of each of the alternative RUNX motifs and RUNX-containing modules, in which the 2 TF motifs are up to 50 bp apart, was determined with Genomatix and denovo motif discovery in Runx3 peaks was carried out using MEME-ChIP [18]. 

### Microarray processing and analysis

RNA and was isolated using the RNeasy Micro kit (Qiagen), according to manufacturer instructions. Purified RNA was reverse-transcribed, amplified and labeled with Affymetrix GeneChip whole transcript sense target labeling kit. Labeled cDNA (3 biological repeats) from WT and Runx3-/- CD8-TC and NKC was analyzed using Affymetrix mouse gene ST 1.0 and A430.2 microarrays, respectively, according to manufacturer instructions. Microarrays were scanned by GeneChip scanner 3000 7G and statistical analysis of data was performed using the Partek® Genomics Suite (Partek Inc., St. Louis, Missouri 63141) software. CEL files (containing raw expression measurements) were imported to Partek GS and data was preprocessed and normalized using the RMA (Robust Multichip Average) algorithm [78] with GC correction or GC-RMA for CD8-TC and NKC, respectively. To identify differentially expressed (DE) genes fold changes were calculated. Gene lists were created by filtering the genes based on a cutoff of 1.5-fold, p-value <0.05 and signal above background in at least one microarray. All microarray and Chip-seq data are available in the GEO public database under accession number GSE50131.

### Statistical and GSEA analyses

The association between 2 variable gene sets, such as differentially expressed genes and Runx3-bound genes, was tested using 2x2 contingency tables with R commander in R statistical software package. The significance of an overlap between 2 similar gene sets, such as Runx3-regulated genes, was carried as described in [23]. GSEA analysis of the relationship between differentially expressed (Runx3^-/-^ versus WT) and Runx3-bound genes was done according to Subramanian et al., 2005 [29]. All microarray genes of IL-2-activated CD8-TC and NKC were first pre-ranked according to absolute linear fold changes of Runx3^-/-^ versus WT expression values and statistical enrichment of Runx3-bound genes within the ranked list was evaluated.

### Real-time reverse transcriptase quantitative PCR (RT-qPCR)

RNA was purified from IL-2-activated WT and Runx3-/- CD8-TC as described above and an equal amount of RNA from the 3 biological repeats was mixed and reverse transcribed with Omniscript^TM^ RT kit (Qiagen). Two RT cDNA reactions were prepared for each RNA sample and then mixed to reduce variability. qPCR was performed using light cycler 480 (Roch, US) with 480 SYBR Green I master (Roch) with Tm 61°C, using *Actb* as calibrator. Fold change between Runx3-/- and WT expression was calculated using Excel-based REST software. The primers used are listed in table S5. 

## Supporting Information

Figure S1 **Western analysis of Runx3 immunoprecipitation (IP).** Anti-Runx3 antibody (Poly-G), but not non-immune serum (NIS), immunoprecipitated Runx3 (left panel) but not GAPDH (right panel) from whole cell extract (WCE) of spleen CD8-TC. Anti-Runx3 (Poly-G) Immunoprecipitated material was analyzed by Western blotting using monoclonal mouse monoclonal anti-Runx3 or anti-GAPDH antibody.
(TIF)

Figure S2 ***Denovo* motif finding and RUNX-containing modules in Runx3-bound regions of IL-2-activated CD8-TC and NKC.** Top 3 motifs in Runx3 occupied promoter (A) or enhancer (B) regions. (C) Enrichment of RUNX-containing modules in Runx3-bound enhancer regions in IL-2-activated CD8-TC and NKC.
(TIF)

Figure S3 **Scatter plot comparing gene expression of IL-2-activated Runx3^-/-^ vs.** **WT CD8-TC and NKC**.
Red and blue dots mark up- or down-regulated genes, respectively, in Runx3^-/-^ vs. WT cells (1.5-fold). Examples of Runx3-regulated genes are indicated.
(TIF)

Figure S4 **Quantitative RT-PCR analysis of 7 Runx3-regulated genes in IL-2-activated Runx3^-/-^ vs.** **WT CD8-TC**.
The 4 down-regulated and 3 up-regulated genes in Runx3^-/-^ vs. WT showed the same pattern as in the microarray analysis. Data represent mean± SE of two independent assays.
(TIF)

Figure S5 **Ingenuity enriched ontology terms of Runx3-regulated genes common to IL-2-activated CD8-TC and NKC.**
(TIF)

Table S1 **Predicted biological functions of Runx3.**
(DOC)

Table S2 **Microarray differentially expressed genes in Runx3^-/-^ versus WT resting and IL-2-activated CD8-TC and NKC.**
(XLS)

Table S3 **Lists of Runx3-regulated genes in resting and IL-2-activated CD8-TC and NKC.**
(XLS)

Table S4 **Lists of Runx3-regulated genes that are common to CD8-TC and NKC.**
(XLS)

Table S5 **Sequence of primers used for qPCR analysis.**
(DOC)
