# Supplementary figures and images for: Runx3-mediated Transcriptional Program in Cytotoxic Lymphocytes
Source: PLoS One. 2013 Nov 13;8(11):e80467. doi: 10.1371/journal.pone.0080467 (PMC3827420; doi:10.1371/journal.pone.0080467)

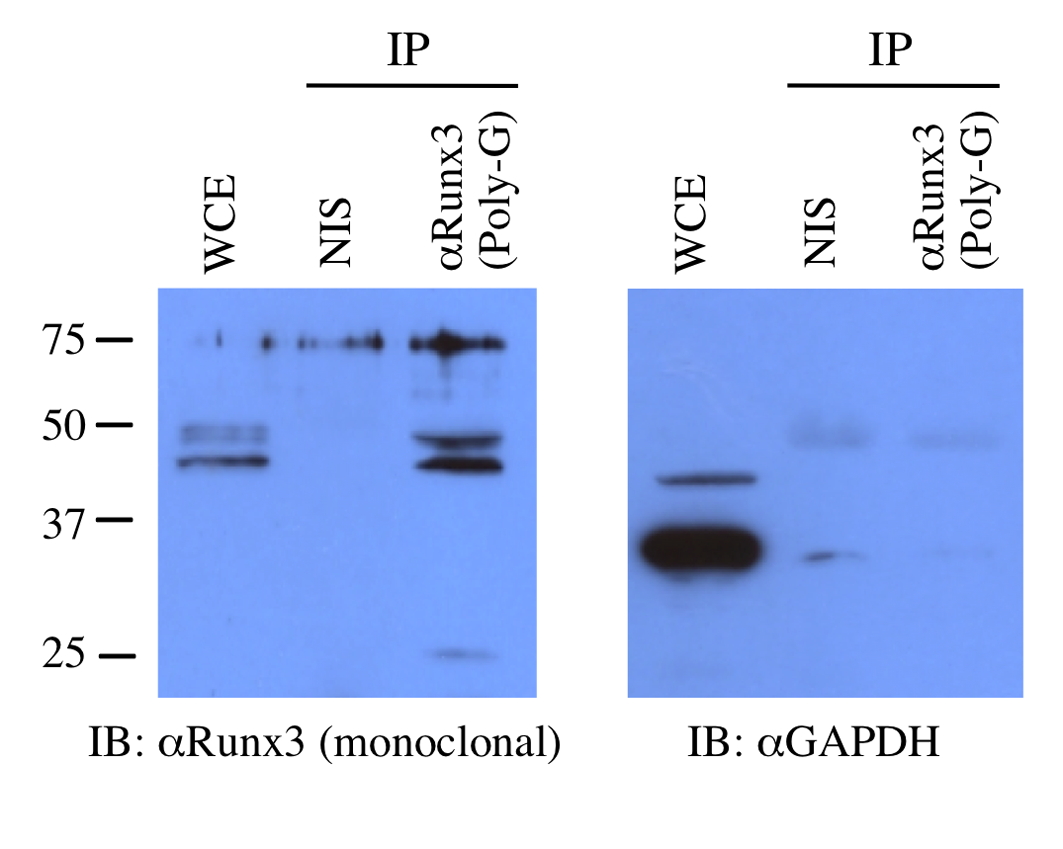

Supplement: Figure S1 — Western analysis of Runx3 immunoprecipitation (IP). Anti-Runx3 antibody (Poly-G), but not non-immune serum (NIS), immunoprecipitated Runx3 (left panel) but not GAPDH (right panel) from whole cell extract (WCE) of spleen CD8-TC. Anti-Runx3 (Poly-G) Immunoprecipitated material was analyzed by Western blotting using monoclonal mouse monoclonal anti-Runx3 or anti-GAPDH antibody. (TIF) [file pone.0080467.s001.tif]

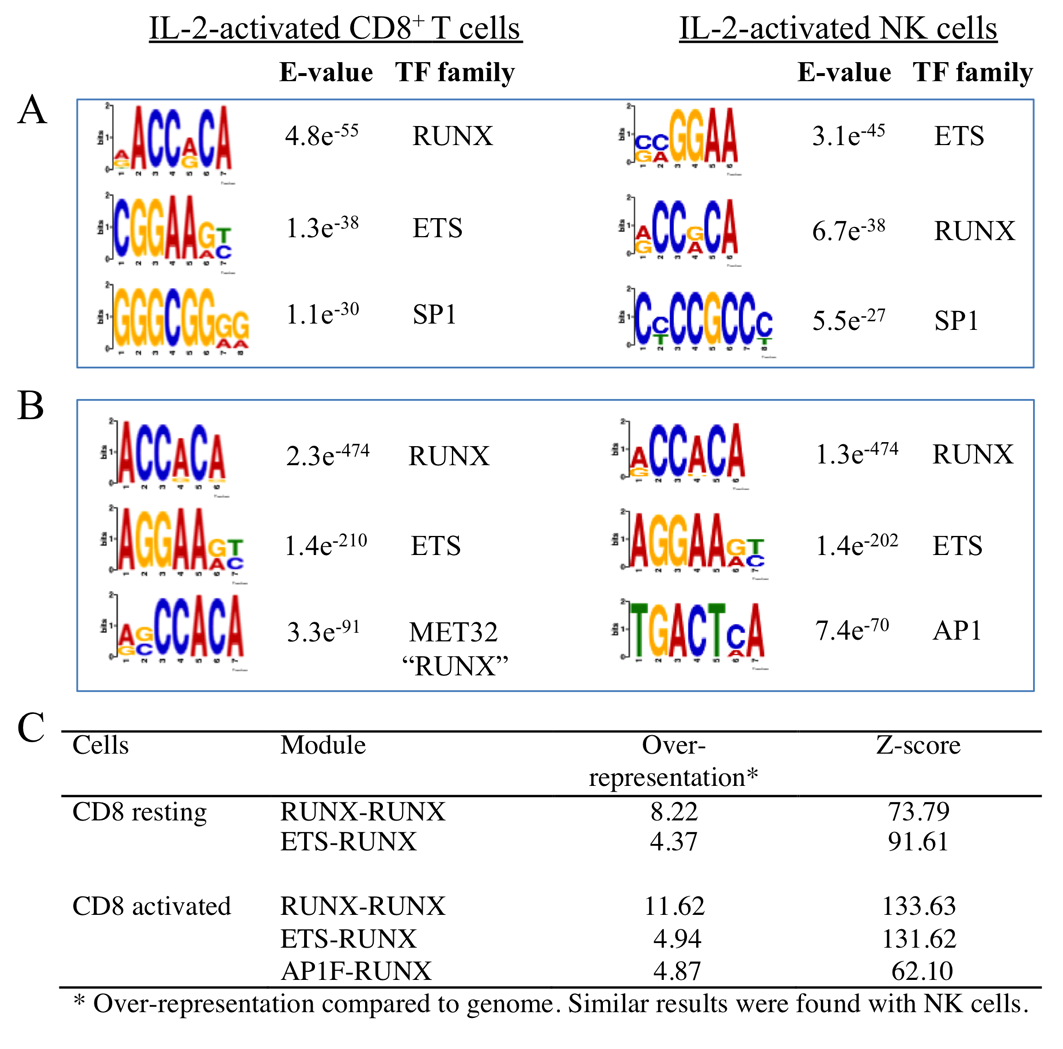

Supplement: Figure S2 — Denovo motif finding and RUNX-containing modules in Runx3-bound regions of IL-2-activated CD8-TC and NKC. Top 3 motifs in Runx3 occupied promoter (A) or enhancer (B) regions. (C) Enrichment of RUNX-containing modules in Runx3-bound enhancer regions in IL-2-activated CD8-TC and NKC. (TIF) [file pone.0080467.s002.tif]

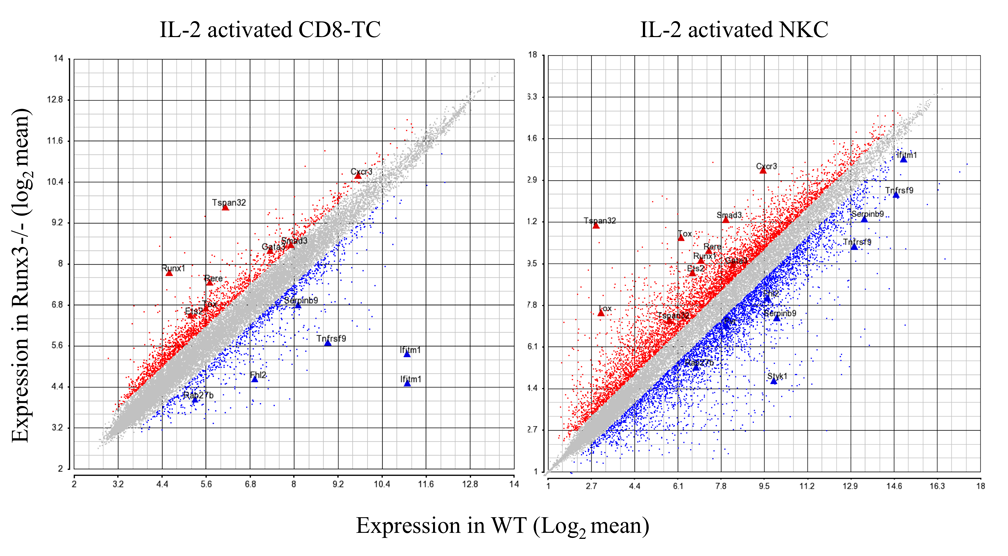

Supplement: Figure S3 — Scatter plot comparing gene expression of IL-2-activated Runx3-/- vs. WT CD8-TC and NKC. Red and blue dots mark up- or down-regulated genes, respectively, in Runx3-/- vs. WT cells (1.5-fold). Examples of Runx3-regulated genes are indicated. (TIF) [file pone.0080467.s003.tif]

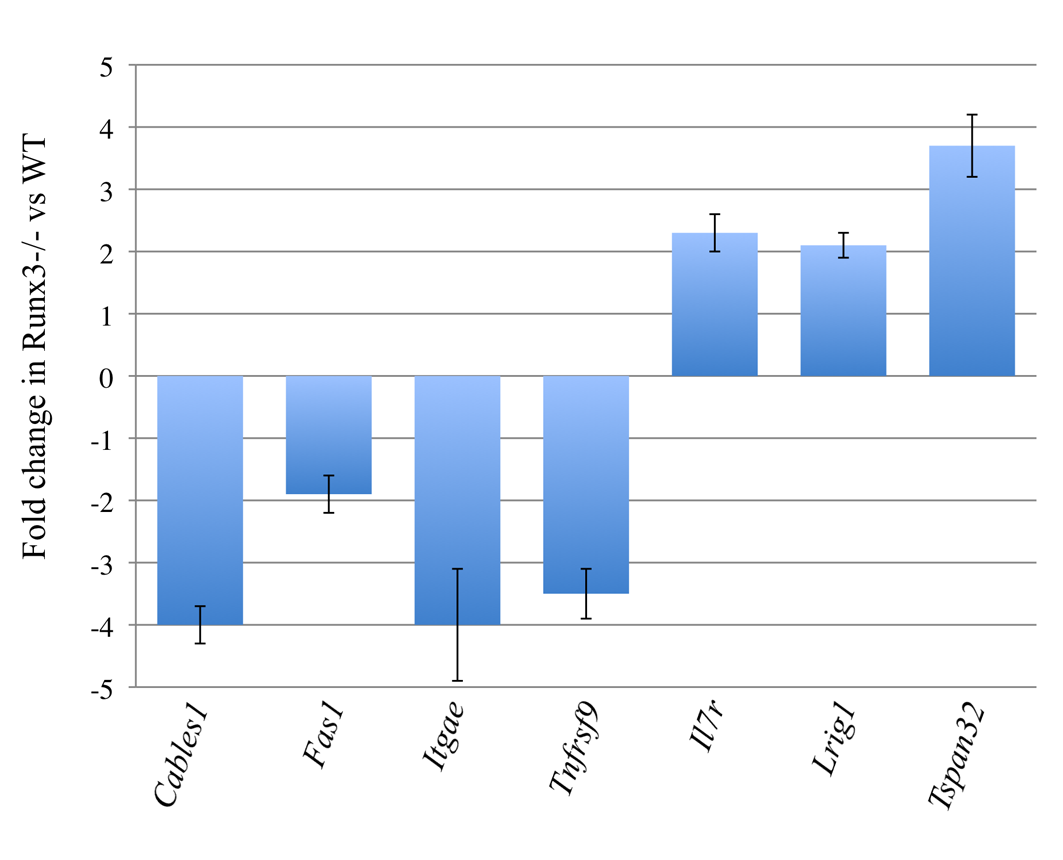

Supplement: Figure S4 — Quantitative RT-PCR analysis of 7 Runx3-regulated genes in IL-2-activated Runx3-/- vs. WT CD8-TC. The 4 down-regulated and 3 up-regulated genes in Runx3-/- vs. WT showed the same pattern as in the microarray analysis. Data represent mean± SE of two independent assays. (TIF) [file pone.0080467.s004.tif]

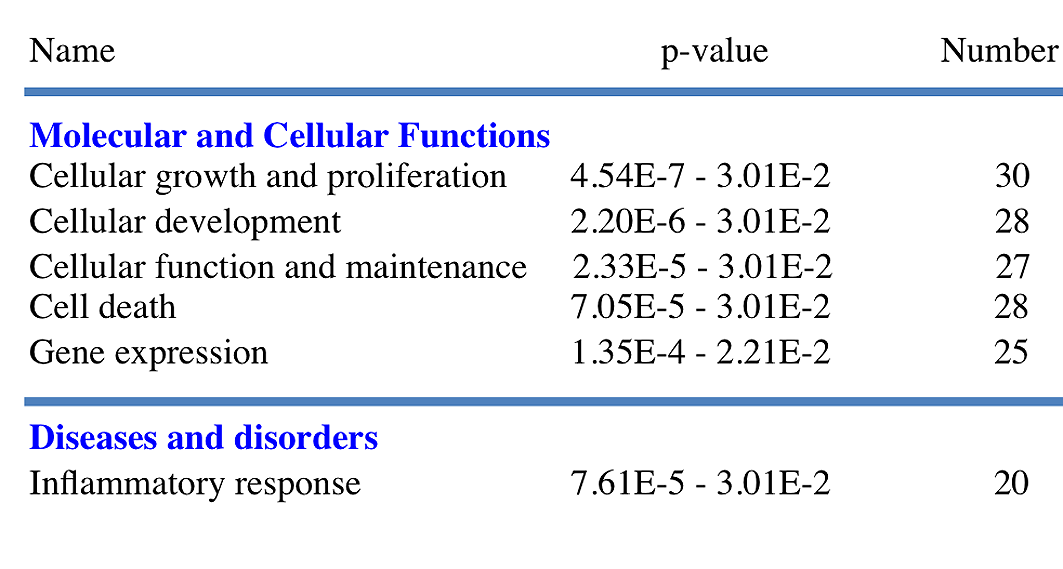

Supplement: Figure S5 — Ingenuity enriched ontology terms of Runx3-regulated genes common to IL-2-activated CD8-TC and NKC. (TIF) [file pone.0080467.s005.tif]
