# Supplementary material for: Runx3-mediated Transcriptional Program in Cytotoxic Lymphocytes
Source: PLoS One. 2013 Nov 13;8(11):e80467. doi: 10.1371/journal.pone.0080467 (PMC3827420; doi:10.1371/journal.pone.0080467)
Supplement: Table S1 — Predicted biological functions of Runx3. (DOC) [file pone.0080467.s006.doc]

**Table S1** Predicted biological functions of Runx3

| Ontology | Term name | Binom FDR q-value | Binom Fold-enrichment | Hyper FDR q-value | Hyper Fold-enrichment |
| --- | --- | --- | --- | --- | --- |
| GO biological process | T cell activation | 2.72e-37 | 2.70 | 1.86e-15 | 2.31 |
|  | Regulation of cytokine production | 7.10e-27 | 2.23 | 2.35e-6 | 1.58 |
|  | Lymphocyte differentiation | 8.68e-27 | 2.38 | 1.35e-10 | 2.10 |
|  | Regulation of immune response | 1.07e-24 | 2.16 | 3.27e-9 | 1.71 |
|  | Immune effector process | 2.48e-22 | 2.57 | 1.95e-3 | 1.52 |
| Mouse phenotype | Abnormal adaptive immunity | 1.63e-116 | 2.12 | 4.58e-44 | 1.73 |
|  | Abnormal T cell physiology | 1.67e-113 | 2.71 | 9.07e-46 | 2.12 |
|  | Decreased lymphocyte cell number | 8.72e-98 | 2.36 | 4.21e-31 | 1.81 |
| Panther pathway | Inflammation mediated by chemokines and cytokine signaling pathway | 1.39e-20 | 2.15 | 1.22e-7 | 1.71 |
|  | T cell activation | 2.62e-11 | 2.44 | 2.24e-5 | 2.07 |
|  | Apoptosis signaling pathway | 3.18e-10 | 2.28 | 3.46e-2 | 1.47 |
|  | Interleukin signaling pathway | 2.99e-8 | 2.16 | 2.94e-2 | 1.54 |
| Pathway commons | TCR signaling in naïve & activated CD8^+^ T cells | 5.90e-49 | 3.82 | 1.25e-20 | 2.76 |
|  | CXCR4 mediated signaling events | 9.14e-46 | 3.09 | 3.21e-19 | 2.38 |
|  | IL-2 mediated signaling events | 6.49e-26 | 2.99 | 8.81e-10 | 2.21 |
| MSigDB pathway | Genes involved in signaling in immune system | 3.37e-28 | 2.12 | 2.51e-11 | 1.78 |
|  | Genes involved in TCR signaling | 5.19e-27 | 3.79 | 7.21e-9 | 2.74 |
|  | Natural killer cell mediated cytotoxicity | 2.19e-18 | 2.83 | 4.62e-6 | 1.98 |
|  | Chemokine signaling pathway | 5.78e-14 | 2.14 | 6.23e-4 | 1.56 |

Runx3-bound regions from resting CD8-TC were analyzed by GREAT. Similar results were obtained with resting NKC and with IL-2-activated CD8-TC and NKC.
