# Supplementary material for: Runx3-mediated Transcriptional Program in Cytotoxic Lymphocytes
Source: PLoS One. 2013 Nov 13;8(11):e80467. doi: 10.1371/journal.pone.0080467 (PMC3827420; doi:10.1371/journal.pone.0080467)
Supplement: Table S5 — Sequence of primers used for qPCR analysis. (DOC) [file pone.0080467.s010.doc]

**Table S5**. **Sequence of primers used for qPCR analysis**

| **Gene name** | **Sequence - 5’ - 3’** |
| --- | --- |
| *Cables1* | F-ttttccgtgctgccgtatcg  R-gttccacaccttcaaggcca |
| *Fasl* | F-tccgtgagttcaccaaccaaa  R-gggggttccctgttaaatggg |
| *IL7r* | F-ggatgggatcctgtcttgcc  R-gatcggggagactaggccat |
| *Itgae* | F-cctgtgcagcatgtaaaagaatg  R-caaggatcggcagttcagatac |
| *Lrig1* | F-ttgaggacttgacgaatctgc  R-cttgttgtgctgcaaaaagagag |
| *Tnfrsf9* | F-cgtgcagaactcctgtgataac  R-gtccacctatgctggagaagg |
| *Tspan32* | F-tgcgctattgggccttcttatg  R-caccaggatgcagaatgacag |
| *Actb* | F-ggctgtattcccctccatcg  R-ccagttggtaacaatgccatgt |
